# Effect of package insert language on health-care providers’ perceptions of influenza vaccination safety during pregnancy

**DOI:** 10.1016/S2214-109X(16)30182-6

**Published:** 2016-10

**Authors:** Karina A Top, Catherine Arkell, Heather Scott, Shelly A McNeil, Jaelene Mannerfeldt, Justin R Ortiz, Philipp Lambach, Noni E MacDonald

**Affiliations:** Department of Pediatrics (KAT, NEM), Department of Community Health and Epidemiology (KAT), Department of Obstetrics and Gynecology (CA, HS), and Department of Medicine, Dalhousie University, Halifax, NS B3H 4R2, Canada (SAM); Canadian Center for Vaccinology, Dalhousie University, IWK Health Centre, Nova Scotia Health Authority, Halifax, NS, Canada (KAT, SAM, NEM); Department of Obstetrics and Gynecology and Department of Family Medicine, University of Calgary, Calgary, AB, Canada (JM); and Initiative for Vaccine Research, World Health Organization, Geneva, Switzerland (JRO, PL)

Despite national and international recommendations that support influenza immunisation in pregnant women, global adoption of these programmes is inadequate.^1^ Reviews by public health experts of inactivated influenza vaccines have not identified safety concerns in pregnant women or their offspring.^2^ These reviews were based largely on non-product-specific data and observational studies because data from product-specific, randomised controlled trials in pregnant women are scarce. However, clinical trial data are the basis for the language of the vaccine product information and package inserts approved by regulatory authorities regarding indications, safety, and use in specific populations.^3^ Pregnancy is not a contraindication for use of most seasonal inactivated influenza vaccines prequalified by WHO for procurement by UN agencies.^4^ However, the WHO Strategic Advisory Group of Experts on Immunization raised concerns that overly precautionary language in package inserts regarding vaccine safety in pregnancy could contribute to hesitancy.^5^ We sought to evaluate the effect of the package insert language on the perceptions of providers of maternal health care on vaccine safety and use in pregnant women.

We recruited health-care providers at two international maternal health conferences and from non-vaccine-related teaching programmes in Ethiopia, Ghana, Uganda, and Laos from Sept 11 to Oct 31, 2015. Individuals who provided health care to pregnant women were invited to complete a ten-item questionnaire in English, French, or Spanish. Following verbal consent, the questionnaire was provided using a tablet computer via an online survey platform. Printed questionnaires (in English) were used at the teaching programme sites.

We developed the questionnaire to capture demographics and perceptions of vaccine safety and use by pregnant women after respondents read three different package insert statements for equivalent WHO pre-qualified seasonal inactivated influenza vaccines. A negatively framed statement emphasised uncertainty about safety and effectiveness: “safety and effectiveness in pregnancy is not established… [use] only if clearly needed” (Fluzone, Sanofi Pasteur, Swiftwater, PA, USA). Two more positively framed statements emphasised conditions for vaccine use: positively framed statement A: “use only following the advice of a healthcare professional, based on consideration of the benefits and risks to the mother and foetus” (FluLaval, GlaxoSmithKline, Sainte-Foy, QC, Canada), and even more positively framed statement B: “use only from the second pregnancy trimester onwards… [use throughout pregnancy in women] at risk of complications of infection” (Vaxigrip, Sanofi Pasteur, Lyon, France).^4^ After reading each statement, respondents indicated how safe they thought the vaccine was on a Likert scale (moderately/very safe, neutral, moderately/very unsafe, don’t know). They were then asked whether they would recommend this vaccine if it was recommended by national health authorities (yes, no, don’t know/no response), and whether the statements would affect what they told pregnant women about immunisation (yes, no, don’t know/no response).

We used Opinio survey software version 6.9.1, which was hosted on a computer server in Halifax, NS, Canada. We used SAS version 9.4 (SAS Institute, Cary, NC, USA) for the analysis. The IWK Health Centre Research Ethics Board and WHO Research Ethics Review Committee approved the study.

We enrolled 141 maternal health-care providers from 49 countries in all six WHO regions; 105 (74%) respondents were from low-income and middle-income countries (LMICs). 24 (17%) participants were recruited from the teaching programmes and 117 (83%) were recruited from the conferences. 112 (80%) respondents were obstetricians, 15 (11%) were midwives or nurses, and 13 (9%) were other professionals. 111 (79%) respondents prescribed or administered vaccines to pregnant women. 106 (75%) respondents read vaccine package inserts occasionally, often, or for new products. Respondents from LMICs were significantly more likely than those from high-income countries (HICs) to read package inserts (80% [84 of 105] *vs* 61% [22 of 36], p=0·02).

Responses to the package insert statements, stratified by country income level, are shown in the figure. After reading the negatively framed package insert statement, 46 (44%) of respondents from LMICs perceived the vaccine described in the statement as unsafe and 26 (25%) perceived it as safe (figure 1A). After reading positively framed statement B, 31 (30%) of respondents from LMICs perceived the vaccine as unsafe and 56 (53%) perceived it as safe. Most respondents from LMICs (83, 79%) and HICs (20, 56%) indicated that the package insert statements would affect how they counselled pregnant women about immunisation (p=0·02; figure 1C). Responses to the package insert statements did not differ by WHO region or profession (data not shown).

The findings suggest that health-care providers perceive package insert information as contradicting WHO and national immunisation recommendations, and that this perceived disagreement could affect their decisions to recommend immunisation to pregnant women. Although the study was limited by the convenience sampling approach, which precluded calculation of the response rate and might have introduced selection bias, the similarities in responses between participants from HICs and LMICs suggest that package insert language can raise safety concerns in many settings.

Regulatory authorities, manufacturers, and public health organisations should work towards reconciling the perceived disagreement between their respective documents and developing a language that is unambiguous to health-care providers. Research is needed to determine the optimal package insert content and language that is readily understood by health-care providers and that facilitates appropriate, evidence-based use of vaccines. Reproducing national or WHO recommendations for vaccine use during pregnancy in the package insert, when they are aligned with the product’s safety profile, could provide health-care workers with specific guidance. Such measures might help to improve vaccine uptake in pregnancy.

## Figures and Tables

**Figure:**
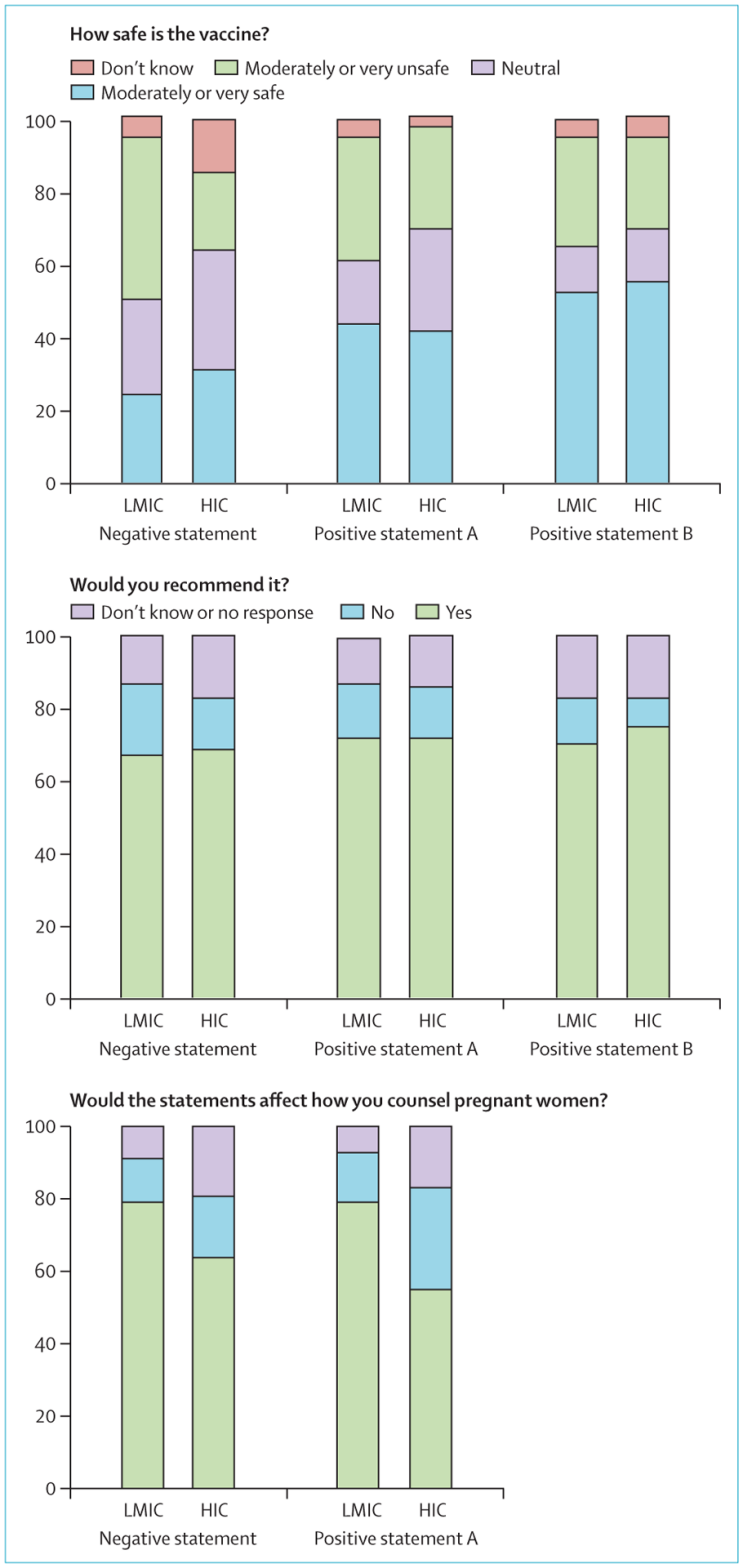
Perceptions of negatively and positively framed precautionary statements about vaccine use during pregnancy Positive statement B not assessed in the final question. LMIC=low-income and middle-income countries (n=105). HIC=high-income countries (n=36). *p=0·02.
